# National-level estimation of long-term economic burden for lung cancer patients in China

**DOI:** 10.3389/fpubh.2026.1844775

**Published:** 2026-09-07

**Authors:** Chengyao Sun, Wenxin Yan, Wude Guo, Yang Liu, Guoxiang Liu

**Affiliations:** 1 Department of Health Economics, College of Health Management of Harbin Medical University, Harbin, China; 2 Department of Outpatient Administration, The Fourth Hospital of Harbin Medical University, Harbin, China; 3 Department of Health Education, College of Public Health of Harbin Medical University, Harbin, China

**Keywords:** China, cost of illness, direct costs, economic burden, indirect costs, long-term, lung cancer

## Abstract

**Background:**

With the rapid increase in the incidence and mortality of lung cancer, a growing number of patients with lung cancer and their families are facing a tremendous economic burden owing to the high cost of treatment in China. This study aimed to estimate the long-term (5 years post-diagnosis) economic burden on patients with lung cancer newly diagnosed in 2015 in China.

**Methods:**

This study utilized data from a multicenter survey, involving 469 patients in eastern, central, and western China. From the survey, direct and indirect economic burdens were calculated, and the number of survivors 5 years later based on the national incidence of lung cancer and the observed survival rate in 2015 were estimated. Based on this, we performed descriptive statistical analysis using Excel and calculated the long-term economic burden on patients with lung cancer across the country in 2015 during the 5 years after disease onset.

**Results:**

The total economic burden for the 2015 cohort 5 years post-diagnosis was $12.7 billion, equivalent to 0.13% of China's 2015 gross domestic product. Direct costs accounted for 64% ($8.1 billion), while indirect costs accounted for 36% ($4.6 billion). Among the investigated components, direct medical costs constituted the largest share (57% of the total burden), followed by indirect costs from missed work (26%), indirect costs from premature death (10%), and direct non-medical costs (7%).

**Conclusion:**

The economic burden of lung cancer in China is substantial and is dominated by direct medical costs. There is an urgent need for policy measures focusing on multi-tiered medical insurance system improvements, cost-control mechanisms, and the promotion of early screening and diagnosis to alleviate this burden on patients, families, and the healthcare system.

## 1 Background

For several decades, lung cancer has been a major public health concern in many countries worldwide. According to the Global Cancer Statistics 2022 released by the International Agency for Research on Cancer of the World Health Organization, lung cancer is the most common cancer worldwide, with 2.48 million cases in 2022 (accounting for 12.4% of all cancer cases). It also remains the leading cause of cancer deaths, with an estimated 1.81 million deaths (18.7% of all cancer deaths) (1).

Notably, lung cancer is a malignant tumor that poses a serious threat to the health of the Chinese population. Data on the incidence and mortality of malignant tumors in China from 2022, released by the National Cancer Center of China in 2024, showed that lung cancer ranked first in terms of both the incidence and mortality of malignant tumors in both men and women (1). There were 1.06 million new cases of lung cancer, accounting for 22.0% of all malignant tumors, and 733,300 deaths, accounting for 28.5% of all malignant tumor-related deaths.

The high incidence and mortality rates of lung cancer impose a severe disease burden, with the associated economic burden on patients and families deserving particular attention. By estimating the global economic cost of cancer from 2020 to 2050 (2), scholars substantiated that the five cancers with the highest costs are tracheal, bronchial, and lung cancers (15.4%); colon and rectal cancer (10.9%); breast cancer (7.7%); liver cancer (6.5%); and leukemia (6.3%). The projected total economic burden of lung cancer will increase to 53.4 billion USD by 2030, accounting for 0.146% of China's gross domestic product (GDP) (3). With an increase in the incidence and mortality of lung cancer across China, the economic burden imposed by the disease has contributed to the growing prominence of poverty stemming from illness in recent years. Therefore, itis critical to investigate the economic burden of lung cancer (3).

Studies have been conducted on the economic burden of lung cancer in China. For instance, Pan calculated the direct economic burden per capita for lung cancer in the central and western regions of China from 2012 to 2018 using medical record data of patients in stages I-IV (4). Wei extracted the medical record data of hospitalized patients with lung cancer from 2015 to 2020 to analyze the growth and changing trends of direct medical expenses for hospitalized patients (5). In addition, most current studies focus on the data of patients who have been ill for 1 year, thus a lack of long-term economic burden research on patients with lung cancer (6, 7). Given that the 5 year survival rate for lung cancer is 19.7% (8), the short-term economic burden of the disease, especially the 1 year direct economic burden, does not fully reflect the main economic burden of the disease for diagnosed patients. Second, the indirect economic burden in most studies only considers the losses from missed work of patients and their family caregivers and rarely considers the economic losses caused by the premature death of patients (9). This underestimates the economic burden of lung cancer.

Given the limitations of the current research, it is necessary to comprehensively measure the long-term economic burden of lung cancer. This study aimed to calculate the economic burden of patients newly diagnosed with lung cancer in China in 2015 over the 5 years following their diagnosis. The findings are aimed to develop a scientific basis for reducing the economic burden of lung cancer.

## 2 Methods

### 2.1 Data collection

A multi-center cross-sectional survey was conducted 2 years after the patient was diagnosed with lung cancer. Based on the National Bureau of Statistics of China economic development classification, the surveyed geographical areas/provinces were categorized as eastern, central, and western regions. Patients with cancer initially diagnosed by professionals between 2015 to 2016 were included. Eligible participants were identified from hospital records and approached for the survey. Data were collected through face-to-face interviews using a structured questionnaire. The interviewers received training and were required to verify the completeness of the questionnaire before concluding each interview. The questionnaire collected data on demographic characteristics, diagnosis and treatment, and treatment related costs. In total, 469 patients with lung cancer were included in this study. The inclusion criteria were as follows: (1) a first-time diagnosis of primary lung cancer, and (2) an initial diagnosis in 2015.

The data collection tools comprised three parts: (1) socio-demographic characteristics (e.g., gender, age, residence, marital status, educational attainment, occupation type, and health insurance); (2) cancer diagnosis and treatment (such as hospital type and completion status of treatment); and (3) cost information in 2015 and 2016 (direct medical costs and direct non-medical costs) and labor loss in 2015 and 2016 (days of missed work for patients and caregivers after the patient was diagnosed).

### 2.2 Cost estimation

The economic burden of a disease refers to the total economic cost and resource consumption due to diseases, disabilities, and premature deaths. In this study, the economic burden included the direct economic burden (direct medical and non-medical costs) and indirect economic burden (economic losses due to missed work and premature death) (10, 11).

#### 2.2.1 Direct economic burden of lung cancer

The direct economic burden of lung cancer includes direct medical and non-medical costs associated with the disease. In this study, direct medical costs included expenditures for medical services and drugs related to the diagnosis and treatment performed at hospitals, clinics, and pharmacies. Direct non-medical costs included transportation, accommodation, meals, additional nutrition, and hiring caregivers during treatment.

**The direct economic burden was calculated as follows:**


$$\begin{array}{l} \text{Direct economic burden in China }=\;\sum \left(\text{DE}{\text{B}}_{\text{i}}\;\times \;{\text{N}}_{\text{i}}\right) \end{array}$$ (1)

Where, i is the year of illness (2015–2020), N_i_ is the remaining survivors in year i of patients with lung cancer who were newly diagnosed in 2015, and DEB_i_ is the direct economic burden per capita.

**The specific measurement steps were as follows:**

First, we calculated the direct medical and non-medical costs in the first and second years after diagnosis using sample data obtained from a cross-sectional survey. Second, from the second year onwards, if the patient's condition does not show significant progression, the treatment plan will shift to a standardized maintenance therapy, and the range of fluctuations in annual treatment costs will be relatively small. Therefore, we assumed that the direct medical and non-medical costs per capita in the third, fourth, and fifth years after diagnosis were equal to those in the second year. Third, we multiplied the direct economic burden per capita for each year by the number of survivors to obtain the annual national direct economic burden. Fourth, we summed the direct economic burden for each year to obtain China's total direct economic burden.

**Calculation method for the number of lung cancer survivors was as follows:**

The number of new cases in 2015, obtained from the China Cancer Registration Annual Report (2017), was 189,052 (12). The 1–5 years observed survival rates of the patients, sourced from published studies in China, were 56.3%, 36.3%, 27.8%, 22.6%, and 20.1%, respectively (13).

#### 2.2.2 Indirect economic burden of lung cancer

The indirect economic burden included lost productivity due to missed work by patients and their family caregivers and premature patient death.

**The indirect economic burden caused by missed work was calculated as follows:**


$$\begin{array}{l} \text{IE}{\text{B}}_{\text{iMW}}\;=\;{\text{G}}_{\text{i}}÷365\;\times \text{ D} \end{array}$$ (2)

Where, IEB_iMW_ represents the indirect economic burden per capita resulting from missed work due to illness. G_i_ is GDP per capita (14) divided by 365 to calculate the daily national income per capita. D denotes the total number of missed work days due to illness, including days spent providing care for patients, as obtained from the above cross-sectional survey. We assumed that D in the third, fourth, and fifth years after diagnosis was equal to that in the second year. Disability-adjusted life years (DALYS) were replaced by days of lost work because DALYs only consider the loss of disability of the patient and do not consider the economic loss of lost work of the patient's family due to caring for the patient.


$$\begin{array}{l} \text{Indirect economic burden caused by missed work in China}\\ \;=\;\sum \left({\text{N}}_{\text{i}}\;\times \;IEB_{\text{iMW}}\right) \end{array}$$ (3)

We added the indirect economic burden caused by missed work in China from 2015 to 2020 to obtain the total indirect economic burden caused by missed work of the patients with lung cancer in China.

Moreover, we calculated the loss of life to estimate the economic burden of premature death.

**Loss of life due to premature death was calculated as follows:**


$$\begin{array}{l} YLL_{\text{i}}=N_{\text{i}}\times L_{\text{i}} \end{array}$$ (4)

Where, YLL_i_ is the years of life lost due to premature mortality, N is the number of deaths, and L is the standard life expectancy at death.

First, the annual number of deaths was calculated based on the number of survivors. Second, the average age of onset of patients in 2015 was calculated using a weighted method, according to the incidence in registration areas of China in 2015 [from the Annual Report of China Tumor Registration (2017 edition)] and the population of China by age group in 2015 (2017 Statistical Yearbook of China Health and Family Planning) (10, 15). The results indicated that the average age of onset for patients newly diagnosed with lung cancer in 2015 was 65 years. Third, the average age at death was presented for each subsequent year. Finally, the YLLs were calculated according to the standard life expectancy (76.3 years) reported by the National Health Commission in 2015 to obtain L. In this calculation, L represents the number of years of life lost per death, obtained by subtracting the age at death from the standard life expectancy.


$$\begin{array}{l} \text{Indirect economic burden due to premature death in China}\\ \;=\;\sum \left({\text{G}}_{\text{i}}\;\times \;{\text{YLL}}_{\text{i}}\;\times \;\text{PW}\right) \end{array}$$ (5)

The productivity weight (PW) of patients aged > 60 years was 0.1. To obtain the total indirect economic burden of premature death in China, the indirect economic burden of premature death in China from 2015 to 2020 was summed.

Cost data were converted into US dollars at an exchange rate of 6.23 yuan per dollar (the average annual exchange rate in 2015 from the China Foreign Exchange Trade System). All expenditure data were discounted until 2020 at a 3% rate.

## 3 Results

### 3.1 Sample characteristics

A total of 469 patients with lung cancer were included in this study. Of the study participants, 73.8% were older adult individuals aged 60 years and above. Among them, 296 (63.1%) were men, and 173 (36.9%) were women. Most patients were married (86.6%), 288 (61.4%) had junior high school education or below, 148 (31.6%) were retired, and more than half of the patients lived with less than or equal to three family members (59.3%). All 469 patients included in the study were enrolled under medical insurance, of whom 54.6% had basic medical insurance for urban employees. Moreover, a total of 83.2% of patients had a disease duration of 2 years (Table 1).

**Table 1:** Participant characteristics.

Characteristics	Number	Percent (%)
Gender		
Male	296	63.1%
Female	173	36.9%
Age (years)		
≤ 60	123	26.2%
>60	346	73.8%
Education level		
Junior high school or below	288	61.4%
High school	111	23.7%
University and above	70	14.9%
Insurance		
UEBMI	256	54.6%
U&RRBMI	207	44.1%
Others	6	1.3%
Occupation		
Employees	102	21.7%
Farmer	149	31.8%
Retiree	148	31.6%
Others	70	14.9%
Marital status		
Married	406	86.6%
Others	63	13.4%
Household size		
≤ 3	278	59.3%
>3	191	40.7%
Course of disease		
1 year	79	16.8%
2 years	390	83.2%

The UEBMI represents urban employee basic medical insurance. U&RRBMI includes resident basic medical insurance (URBMI) and the new cooperative medical scheme (NCMS).

### 3.2 Economic burden of lung cancer

#### 3.2.1 Direct economic burden of lung cancer

##### 3.2.1.1 Direct economic burden of participants

In the cross-sectional survey, 469 patients were then divided into the following groups: those who completed the treatment and those who did not (Table 2). The main reasons for patients not completing the full course of treatment included death (37.5%) and inability to treat severe conditions (21.9%) (Table 3). In the first year of diagnosis, 93.2% (437 people) completed full treatment; the direct economic burden per capita of these patients was $17,273.8, and the direct medical cost per capita was $15,642.2. The direct non-medical costs per capita were $1,631.7, accounting for 9.4% of the total costs. Medical insurance accounted for 44.9% of direct medical costs. The top three direct non-medical costs per capita were meals, nutritional supplements, and accommodation expenses, accounting for 23.6%, 22.9%, and 18.6%, respectively.

**Table 2:** Direct economic burden of participants.

		Cost per patient of first year of diagnosis ($) (*N*=469)		Cost per patient of second year of diagnosis ($) (*N* = 390)	
Cost categories	Cost items	The patient completed treatment (*N* = th437)	The patient uncompleted treatment (*N* = th32)	The patient completed treatment (*N* = th361)	The patient uncompleted treatment (*N* = th29)
Direct medical costs		15,642.2	9,429.8	9,581.8	7,330.0
	Medical insurance	7,029.1	3,115.0	4,268.4	3,086.3
	Out of pocket	8,613.0	6,314.8	5,313.3	4,243.7
Direct non-medical costs		1,631.7	1,349.3	1,323.3	1,282.3
	Transportation	302.4	271.8	267.0	131.2
	Accommodation	302.7	139.2	218.5	114.9
	Meals	385.5	183.3	320.0	272.3
	Nutrition	374.4	335.4	405.6	396.0
	Hired attendant	86.3	104.2	35.4	97.8
	Others	180.5	315.4	76.8	270.0
Direct economic burden		17,273.8	10,779.1	10,905.0	8,612.3

**Table 3:** Reasons for incomplete treatment.

Reasons	Reasons for incomplete treatment (the first year of diagnosis)	Reasons for incomplete treatment (the second year of diagnosis)
Economic hardship	5 (15.6%)	7 (24.1%)
The patient's condition is too severe to be treated	7 (21.9%)	4 (13.8%)
The patient voluntarily gave up treatment	4 (12.5%)	2 (6.9%)
Death	12 (37.5%)	12 (41.4%)
Other	4 (12.5%)	4 (13.8%)
Total	32 (100%)	29 (100%)

Among the 469 respondents, 390 still had expenses in the second year of treatment, and 92.6% completed the full course of treatment. The direct economic burden per capita for these patients was $10,905, which was lower than the cost incurred in the first year. Direct medical expenses ($9,581.8) and direct non-medical expenses ($1,323.3) per capita accounted for 87.9% and 12.1%, respectively. The top three indirect medical expenses were related to nutrition, meals, and transportation (Table 2). The main reasons for incomplete treatment in the second year were death (41.4%) and economic hardship (24.1%) (Table 3).

##### 3.2.1.2 Direct economic burden in China

Based on the direct economic burden per capita obtained from the cross-sectional survey, we calculated the direct economic burden of the remaining new cases in China in 2015. The total direct cost to patients with lung cancer over the years since diagnosis was $8.1 billion (Table 4).

**Table 4:** Direct economic burden in China.

Period	Survival	Direct economic burden (million,$)	Discounted direct economic burden (million,$)
The first year	189,052	3,265.6	4,136.8
The second year	106,436	1,160.7	1,427.5
The third year	68,626	748.4	893.6
The fourth year	52,556	573.1	664.4
The fifth year	42,725	465.9	524.4
The Sixth year	37,999	414.4	452.8
Total		6,628.1	8,099.5

#### 3.2.2 Indirect economic burden

##### 3.2.2.1 Indirect economic burden of participants

Among the 469 patients surveyed, the number of missed days of work per patient and caregiver in the first year of diagnosis was 165 and 82 days, and 183 and 54 days in the second year, respectively. The indirect economic burden per capita in the first and second years after diagnosis was $4,800 and $5,000, respectively (Table 5).

**Table 5:** Indirect economic burden of participants.

Indirect economic burden	The first year of diagnosis	The second year of diagnosis
Days of missed work for patients	165	183
Days of missed work for accompanying relatives	82	54
National per capita GDP ($)	7,092.2	7,659.6
Indirect economic burden ($)	4,800	5,000

##### 3.2.2.2 Indirect economic burden in China

The indirect economic burden of new cases in 2015 included the burden caused by missed work and premature death. In the 6 years since the diagnosis of these patients, the indirect economic burden caused by missed work was $266.6 million, and the indirect economic burden caused by premature death was $1,177.6 million. The total indirect economic burden was $4,630.6 million (Table 6).

**Table 6:** Indirecteconomic burden of lung cancer patients in China.

Years	Survival	The indirect economic burden per capita caused by missed work ($)	The indirect economic burden caused by missed work (million, $)^a^	Deaths (*N*)	Age at death	Life expectancy (L)	YLLs	Per capita GDP ($)	The indirect economic burden due to premature death (million, $)^b^	The total indirect economic burden (million, $)^c^	Total indirect economic burden discounted (million, $)
The first year	18,9052	4,822.7	911.7	0	65	11.3	0	7,092.2	0	911.7	1,155
The second year	106,436	4,964.5	528.4	82,616	66	10.3	850,944.8	7,659.6	651.8	1,180.2	1,451.5
The third year	68,626	5,531.9	379.6	37,810	67	9.3	35,1633	8,510.6	299.3	678.9	810.6
The fourth year	52,556	6,099.3	320.6	16,070	68	8.3	133,381	9,361.7	124.9	445.4	516.4
The fifth year	42,725	6,383	272.7	9,831	69	7.3	71,766.3	9,929	71.3	344	387.1
The Sixth year	37,999	6,666.7	253.3	4,726	70	6.3	29,773.8	10,212.8	30.4	283.7	310
Total	497,394		2,666.4						1,177.6	3,844	4,630.6

^*^c = a + b. ^*^The total indirect economic burden = the indirect economic burden caused by missed work + the indirect economic burden due to premature death.

#### 3.2.3 Total economic burden

**Figure 1:**
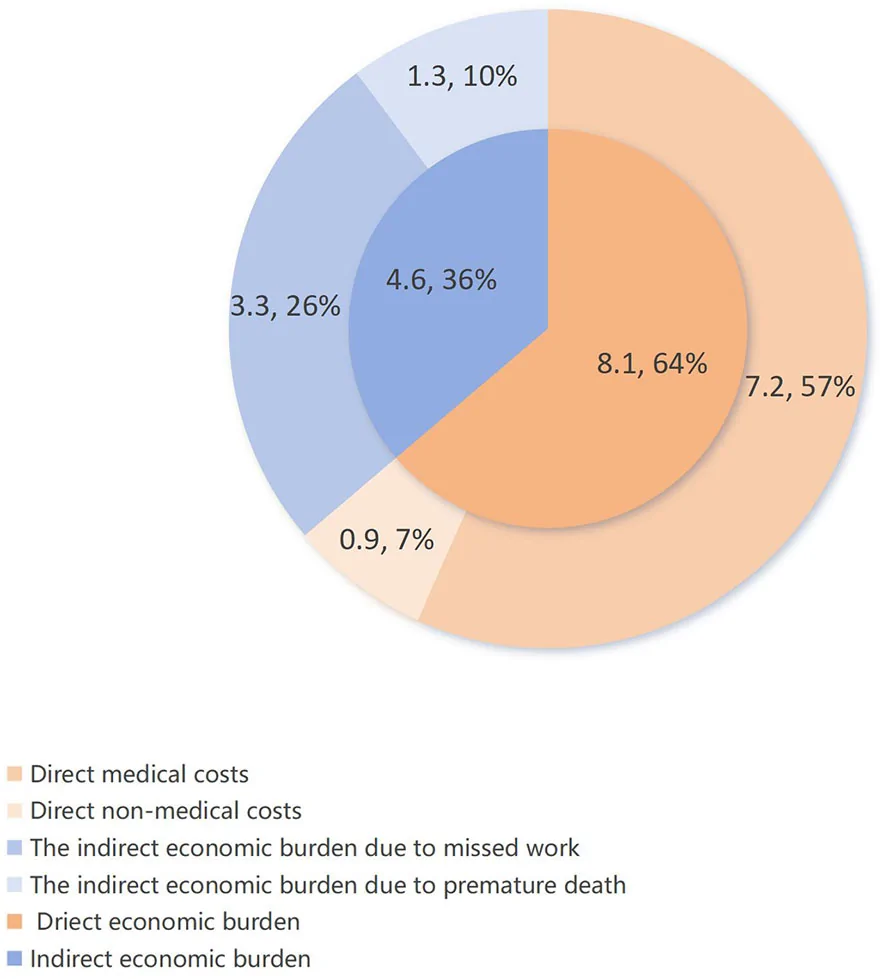
Total economic burden of lung cancer in China (billion, $).

By combining the direct and indirect economic burdens calculated above, the total economic burden of patients with lung cancer in China in 2015 within 5 years after diagnosis was estimated at $12.7 billion, of which 64% was direct ($8.1 billion) and 36% was indirect ($4.6 billion) (Figure 1).

## 4 Discussion

This study aimed to analyze the long-term economic burden (five years after diagnosis) of patients with lung cancer in China in 2015. In the cross-sectional survey, the direct economic burden per capita was $17,273.8, of which direct medical costs accounted for $15,642.2, representing the largest share of the direct economic burden per capita (90.6%). This burden was 2.2 times GDP per capita ($7,092) and five times the disposable income per capita ($3,116) in 2015 (9, 16). Direct non-medical costs per capita were $1,631.7 (9.4% of the direct economic burden), with the top two being nutrition and meal costs, consistent with relevant research results (17, 18). Under the influence of health loss of tumor diseases and adverse reactions of anti-tumor therapy, patients with lung cancer often need timely nutritional intervention to improve body immunity and reduce adverse reactions of treatment.

In view of the high direct medical costs per capita for patients with lung cancer in China, the government should strengthen the coordination among basic medical insurance, serious illness insurance, medical assistance, commercial health insurance, and charitable support systems. Efforts should focus on gradually improving the multi-tiered medical insurance system and ensuring timely reimbursement for drugs and other high-value clinical treatments, thereby reducing direct medical costs for patients.

The indirect economic burden per capita due to missed work was $4,800, accounting for 0.68 times of China's per capita GDP in 2015 ($7,092.2). These results are similar to those reported by Yang et al. (19). Therefore, indirect losses for patients with lung cancer cannot be ignored. In light of this, the government should pay more attention to the indirect economic burden, advocate the expansion of the scope of community nursing services, provide professional escort services for patients with serious diseases such as cancer, include these patients in the scope of long-term care insurance reimbursement, and reduce the economic costs related to escorting (18).

Less than 10% of the patients in our study did not complete the full course of treatment, with the most important reasons being death, lost treatment opportunities, and financial difficulties. At present, the participation rate of basic medical insurance in China continues to stabilize at 95%; however, medical care services covered by basic medical insurance are limited, and the direct medical burden related to lung cancer remains heavy. Moreover, owing to the nonspecific nature of early stage lung cancer, most patients are diagnosed at an advanced stage. Given the poor prognosis of the disease, many patients hope to reduce treatment costs to avoid burdening their families, leading some to forgo treatment (20).

In conclusion, the total economic burden on patients with lung cancer diagnosed in 2015 in China was $12.7 billion, accounted for 0.13% of the national GDP in 2015 (US $9.8 billion), of which 64% was direct ($8.1 billion) and 36% was indirect ($4.6 billion). The proportions of economic burden from highest to lowest were direct medical costs (57%), indirect burden caused by missed work (26%), indirect burden caused by premature death (10%), and direct non-medical costs (7%). In a study conducted in South Korea, the economic burden on patients with lung cancer diagnosed between 2011 and 2015 was estimated to be $9.04 billion (21). Therefore, lung cancer remains a significant cause of cancer economic burden. Direct medical costs account for more than half the economic burden on patients with lung cancer in China. Owing to this, the government should establish a scientific and effective supply side restriction mechanism, improve the payment pattern of medical insurance, strengthen the supervision of medical costs, and encourage medical institutions to standardize their medical behavior and control medical costs. In addition, early screening, diagnosis, and treatment of lung cancer should be actively promoted throughout the country to reduce the incidence of advanced cancer, improve survival, and reduce the economic burden of lung cancer (22, 23).

## 5 Limitations

This study has several limitations. First, our data did not allow us to estimate the costs stratified by lung cancer pathological sub-type and clinical stage of the disease. Second, conducting a multicenter, in-person survey among patients with lung cancer was challenging, hence the sample size was limited. Future studies should aim to include larger samples.

## 6 Conclusions

The economic burden of lung cancer is non-negligible and can have negative effects on individuals, families, and countries. Estimating the long-term economic burden on patients with lung cancer in China provides suggestions for reducing the economic burden of the disease in a targeted manner; positive measures with particular emphasis are urgently needed for all patients with lung cancer to potentially reduce cancer-related economic burden.

## Data Availability

The raw data supporting the conclusions of this article will be made available by the authors, without undue reservation.
